# Fotemustine, teniposide and dexamethasone versus high-dose methotrexate plus cytarabine in newly diagnosed primary CNS lymphoma: a randomised phase 2 trial

**DOI:** 10.1007/s11060-018-2970-x

**Published:** 2018-08-14

**Authors:** Jingjing Wu, Lingling Duan, Lei Zhang, Zhenchang Sun, Xiaorui Fu, Xin Li, Ling Li, Xinhua Wang, Xudong Zhang, Zhaoming Li, Hui Yu, Yu Chang, Feifei Nan, Jiaqin Yan, Li Tian, Xiaoli Wang, Mingzhi Zhang

**Affiliations:** Department of Oncology, Lymphoma Diagnosis and Treatment Centre of Henan Province, The First Affiliated Hospital of Zhengzhou University, Νo. 1 Jianshe East Road, Zhengzhou, 450052 Henan People’s Republic of China

**Keywords:** Teniposide, Methotrexate, Primary CNS lymphoma, Chemotherapy, Whole-brain radiotherapy

## Abstract

**Objective:**

This prospective, randomized, controlled and open-label clinical trial sought to evaluate the tolerability and efficacy of the FTD regimen (fotemustine, teniposide and dexamethasone) compared to HD-MA therapy (high-dose methotrexate plus cytarabine) and to elucidate some biomarkers that influence outcomes in patients with newly diagnosed primary CNS lymphoma.

**Methods:**

Participants were stratified by IELSG risk score (low versus intermediate versus high) and randomly assigned (1:1) to receive four cycles of FTD or HD-MA regimen. Both regimens were administered every 3 weeks and were followed by whole-brain radiotherapy. The primary endpoints were overall response rate (ORR), progression-free survival (PFS) and overall survival (OS).

**Results:**

Between June 2012, and June 2015, 52 patients were enrolled, of whom 49 patients were randomly assigned and analyzed. Of the 49 eligible patients, no significant difference was observed in terms of ORR between FTD (n = 24) and HD-MA (n = 25) groups (88% versus 84%, respectively, *P* = 0.628). Neither the 2-year PFS nor the 3-year OS rate differed significantly between FTD and HD-MA groups (37% versus 39% for 2-year PFS, *P* = 0.984; 51% versus 46% for 3-year OS, *P* = 0.509; respectively). The HD-MA group showed more serious neutropenia (*P* = 0.009) than the FTD group. High Bcl-6 expression correlated with longer OS (*P* = 0.038).

**Conclusions:**

FTD chemotherapy appeared to be safe and effective for PCNSL patients. High Bcl-6 expression correlated with longer survival.

## Introduction

Primary central nervous system lymphoma (PCNSL) is typically an aggressive non-Hodgkin lymphoma, usually of large B-cell histology, with increasing incidence [1]. To date, a standardized approach has not yet been established for the treatment of PCNSL. Combination chemotherapy regimens with HD-MTX followed by whole-brain radiotherapy (WBRT) have been shown to improve survival to a median overall survival (OS) of 30–60 months [2–4]. A phase 2 randomized trial [5] demonstrated that patients given the HD-MA followed by WBRT experienced overall response rate (ORR) of 69% compared to 40% for methotrexate alone (*P* = 0.009), suggesting that polychemotherapy is more effective than single agent HD-MTX. Howerover, increased toxic effects were recorded in the HD-MA protocol. In addition, with prolonged survival times, severe neurological impairment developed after combined treatment, mainly in elderly patients, which mainly attributed to the synergistic toxicity of HD-MTX and WBRT [6]. Given these unsatisfactory outcomes, it is imperative to develop more effective and less toxic innovative therapeutic regimens for PCNSL. Fotemustine is a drug with antitumor activity in disseminated melanoma and primary brain tumors in humans. After systemic administration, fotemustine is largely distributed into the central nervous system [7]. Teniposide, a topoisomerase inhibitor, has been used in the treatment of PCNSL [8, 9], and its penetration in normal brain tissue and in brain tumor tissue has been widely examined [10]. In our previous retrospective study, 16 patients with CNS lymphoma were initially treated with the FTD (fotemustine, teniposide and dexamethasone) regimen. The complete remission (CR) rate was 50%, and the partial remission (PR) rate was 38% [11]. Accordingly, we devised a therapeutic FTD regimen and performed a prospective randomized controlled clinical trial to investigate the efficacy and safety of this treatment compared to HD-MA in patients with newly diagnosed PCNSL. And, we also aimed to detect the expression of biomarkers to explore the correlation between biological markers and the prognosis of PCNSL in a clinical trial setting.

## Methods

### Patients

This randomized, controlled, open-label study compared the efficacy and safety of the FTD regimen with those of HD-MA therapy followed by WBRT in patients with newly diagnosed PCNSL. This trial is registered with ClinicalTrials.gov (NCT01960192). Immunocompetent patients with newly diagnosed PCNSL according to the World Health Organization (WHO) criteria treated at the lymphoma center of the First Affiliated Hospital of the Zhengzhou University between June 2012, and June 2015 were eligible. The diagnosis was established by a neuro-pathological analysis of tumor samples using conventional histology and immuno-histochemistry with CD20, Bcl-6, MUM1, CD10 and MIB-1 staining.

Staging work-up and pre-treatment tests were conducted at the participating center before initiating treatment and included the following: physical examination; mini-mental status examination (MMSE); laboratory work-up of complete blood cell counts; biochemical serum profile; lactate dehydrogenase (LDH); HIV, hepatitis B virus, and hepatitis C virus serological assessments; cytomegalovirus test; contrast-enhanced thorax-abdomen CT scan; testicular ultrasonography (for elderly male patients); gadolinium-enhanced whole-brain MRI; bone marrow biopsy; ophthalmological assessment (including slit-lamp examination); cerebrospinal fluid (CSF) examination (a sample of 2–3 mL of native CSF was obtained for CSF flow cytometry and physicochemical examinations); echocardiography; and respiratory volumes assessment. FDG-PET was optional. For further details on inclusion criteria please see Table 1.

**Table 1:** Inclusion criteria

1. Age range 14–69 years old
2. ECOG performance status 0–2
3. Estimated survival time > 3 months
4. Histologically confirmed PCNSL
5. No previous chemotherapy or radiotherapy
6. No chemotherapy contraindications: hemoglobin ≥ 90 g/dl, neutrophils ≥ 1.5 × 10^9^/L, platelets ≥ 100 × 10^9^/L, aspartate transaminase and alanine transaminase ≤ 2 × ULN, serum bilirubin ≤ 1.5 × ULN, serum creatine ≤ 1.5 × ULN, serum albumin ≥ 30 g/L, and normal serum plasminogen
7. At least one measurable lesion
8. No other serious diseases
9. Normal cardiopulmonary function
10. Negative pregnancy test for women of reproductive age
11. Patients could be followed-up
12. No other relative treatments including traditional Chinese medicine, immunotherapy, and biotherapy, except for symptomatic treatments

*ECOG* Eastern Cooperative Oncology Group, *ULN* upper limitation of normal

### Randomisation and treatment regimen

After staging, they were stratified by International Extranodal Lymphoma Study Group (IELSG) risk score [12] and randomized in a 1:1 ratio based on a computer-generated randomisation schedule to receive either the FTD or HD-MA therapeutic regimen. No-one involved in the study was masked to treatment assignment. The FTD regimen included fotemustine 100 mg/m^2^, 1 h infusion, on day 1; teniposide 60 mg/m^2^, > 0.5 h infusion, on days 2–4; and dexamethasone 40 mg, 1 h infusion, on days 1–5. The HD-MA therapy combined methotrexate 3.5 g/m^2^, 0.5 g/m^2^ in 15 min, followed by 3.0 g/m^2^ in a 6 h infusion on day 1; and cytarabine 1.0 g/m^2^ 1 h infusion, every 12 h, on days 2–3. Patients received adequate hydration, urinary alkalinisation, and folinic rescue before and after methotrexate. Granulocyte colony-stimulation factor (G-CSF) was administered as support when necessary. Intrathecal chemotherapy was not included in the chemotherapy regimens. Chemotherapy was administered according to the condition of the patients. Dosage adjustments should be made during treatments (decrease up to 80% of planned doses) in patients who develop grade 3 or 4 neutropenia or thrombocytopenia to maintain the timing of chemotherapy. Cycles (21 days) were repeated four times, unless there was evidence of progressive disease (PD) or significant toxicity to contraindicate continuation of the treatment. Reasonable delays were allowed.

Efficacy was evaluated every two cycles. The patients in CR and those with a PR after two chemotherapy cycles received two more cycles of the same regimen. After four chemotherapy cycles, patients aged 60 years or younger in CR were treated with 30 Gy consolidating WBRT (30 Gy in 2-Gy fractions × 15) 3–5 weeks after chemotherapy completion; those older than 60 years in CR were treated with watchful waiting. Patients who had PD at any time and patients without CR after four chemotherapy cycles, regardless of age, were referred for rescue WBRT with 30 Gy plus a 15 Gy boost but were kept on study. This study was performed following the Good Clinical Practice Guidelines and the Declaration of Helsinki. This work was approved by the Local Ethics Committee of Zhengzhou University and the Scientific Council of the Faculty of Medicine. All patients were fully informed about the nature and possible toxicities of the treatment protocol and submitted written informed consent.

### Biomarker detection

The assessment of candidate molecular prognostic markers including Bcl-2, Bcl-6, MYC proto-oncogene (c-Myc), melanoma associated antigen (mutated) 1 (MUM-1), CD5 molecule (CD5), membrane metalloendopeptidase (CD10), phosphatase and tensin homolog (PTEN), tumor protein p53 (p53) and marker of proliferation Ki-67 (Ki-67) were performed by immunohistochemistry in archival formalin-fixed paraffin-embedded tissue. Two pathologists, with no knowledge of the clinical data, interpreted the immunohistochemical labeling independently. For PTEN and p53, the degree of staining was graded based on the proportion of positive cells as follows: negative, positive rate < 5%; weakly positive, 5–25%; moderately positive, 25–50%; and strongly positive, > 50%. Ki-67 was quantified by determining the number of positive cells expressing nuclear Ki-67 among the total number of cells within a high-power (×40 objective) microscopic field [13]. We stratified the patients into two subgroups according to the Ki-67 rate with a cutoff value of 90%. For the remaining molecular markers, positivity was assigned if > 30% of the tumor cells showed staining [14]. Bcl-2 staining was considered positive if the staining was either nuclear or cytoplasmic. Only membrane staining was considered positive for the antigen CD10, and nuclear staining was considered positive for Bcl-6 and MUM1/IRF-4.

### Assessment of adverse effects and response criteria

Adverse reactions were monitored by biochemistry and hematological tests, electrocardiograms and routine physical examination. These reactions were graded according to the National Cancer Institute Common Terminology Criteria for Adverse Events, version 3.0 and assessed from the first cycle of the regimen until one month after the terminal treatment [15], and delayed neurotoxicity assessed by clinical examination, and by white matter changes or brain atrophy on MRI or CT [16]. Neurotoxicity analyses were limited to patients who achieved WBRT. Moreover, we excluded patients with cognitive impairment or cerebellar dysfunction at baseline that persisted after study treatment. The response definition was based on changes in the tumor size of enhanced lesions on MRI, which was performed every two cycles and after WBRT, following the modified International PCNSL Collaborative Group (IPCG) Response Criteria [17]. CR was defined as the complete disappearance of all evidence of lymphoma; PR as a 50% or more reduction in tumor size; PD as a 25% or more unequivocal growth in tumor size or development of a new lesion; and SD as situations that did not meet any of the previous criteria. In cases with concomitant positive CSF, cytology examination was performed after the second and fourth cycles of chemotherapy and after treatment completion; a reduction of > 50% of the cell number was considered a PR, whereas a lower reduction was considered SD. Follow-up visits were planned every 3 months during the first year, every 6 months for the following 4 years, and then annually until relapse or the last follow-up.

### Statistical analysis

The primary objectives were to assess the ORR, progression-free survival (PFS) and OS. Several secondary endpoints included assessing toxicity and investigating the immunophenotypic markers influencing outcomes. PFS was defined as the time from diagnosis to disease recurrence, disease progression, or death. OS was defined as the time from diagnosis to death or to the last follow-up. We analyzed the differences between therapeutic groups in response rates with the χ^2^ test. Mann–Whitney tests were used to compare quantitative and ordinal variables. The Kaplan–Meier method was used for the univariate analysis of survival, with the assessment of differences by the log-rank test. Confidence intervals (CI) for survival times were formed, as is standard, from the log transform of the Kaplan–Meier (product-limit) estimator of survival. A multivariate analysis was performed using the Cox proportional hazard model. All *P* values reported were two-sided, and *P* < 0.05 was considered statistically significant. Follow-up was closed for analysis in November 2017. Statistical analyses were performed with SPSS version 21 (SPSS, Inc., Chicago, IL, USA) and GraphPad Prism 6 (GraphPad Software Inc., La Jolla, CA, USA).

## Results

### Baseline characteristics of the patients

**Fig. 1:**
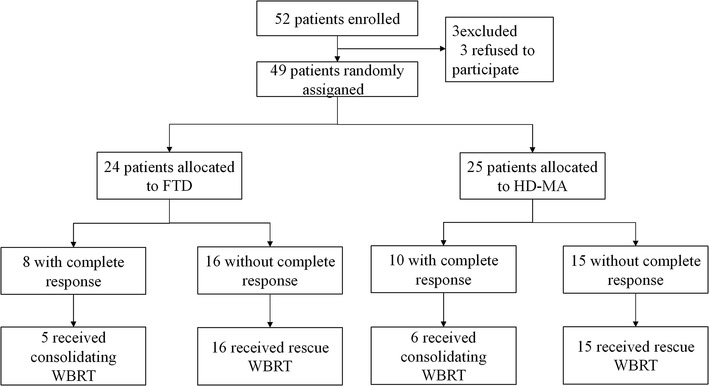
Trial profile

Figure 1 shows the trial profile. 52 patients with PCNSL were recruited. Eventually, of the 49 eligible patients, all patients were diagnosed with diffuse large B-cell lymphomas (DLBCL). All the parameters including age, ECOG performance status, LDH, location of the lesions and CSF protein were available for the 49 patients. 6(12%) patients had concurrent sites of CNS involvement: three intraocular, two leptomeninges, and one spinal cord. The median age at initial diagnosis of PCNSL was 55 years (range 16–69 years), and 30 (61%) patients were male. 33 (67%) patients were aged 60 years or younger, and 23 (47%) patients had elevated LDH levels. 28 (57%) patients had elevated CSF protein levels. Comparative clinical presentations of each arm are listed in Table 2. Patient characteristics were well balanced, and no significant differences were observed between the FTD (24 cases) and HD-MA (25 cases) groups.

**Table 2:** Baseline patient characteristics

Characteristics	Number of patients(n = 49)		
	FTD (n = 24)	HD-MA (n = 25)	*P* value
Median age, years	56 (20–69)	57 (16–67)	0.251*
Gender			
Male	14	16	0.684
Female	10	9	
ECOG PS > 1	14	15	0.906
Elevated LDH	11	12	0.982
Elevated CSF protein	13	15	0.680
Multiple lesions	18	22	0.240

*ECOG PS* Eastern Cooperative Oncology Group performance status, *LDH* lactate dehydrogenase, *CSF* cerebrospinal fluid

*P*: Chi-squared test

*t test

### Response and outcomes

**Fig. 2:**
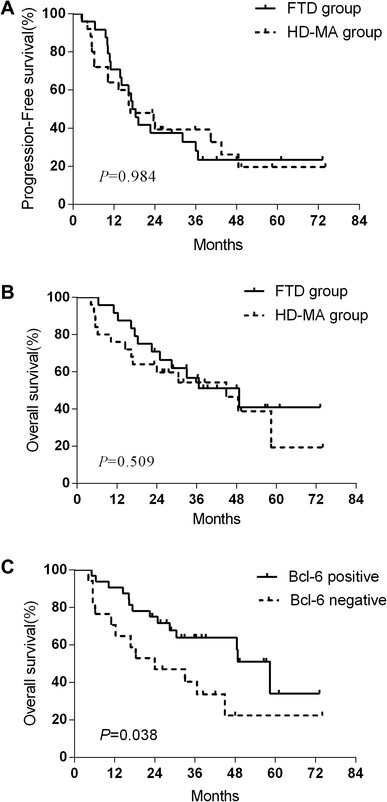
Survival curves. Progression-free survival curves (**a**); overall survival curves (**b**); overall survival curves based on Bcl-6 expression (**c**). The censored observations have been marked in the graphs

All patients were evaluable for response. No major protocol deviations occurred during induction. At the end of chemotherapy, 8 (33%) of 24 patients in FTD group, 10 (40%) of 25 in HD-MA group achieved a CR. 13 patients in FTD group, 11 in HD-MA group achieved a PR, with an overall response rate (complete response plus partial response, ORR) of 88% in FTD group and 84% in HD-MA group (Table 3), whereas no significant difference was detected in terms of the ORR between the FTD group and the HD-MA group (*P* = 0.628). At a median follow-up time of 28.8 months (range 4.2–74.0 months) for eligible patients, the 2-year PFS rate was 37% in the FTD group and 39% in the HD-MA group (*P* = 0.984) with a median PFS of 17.4 months (95% CI 13.9–20.1) versus 16.7 months (95% CI 5.2–28.4), respectively (Fig. 2a). The 3-year OS rate was 51% and 46% in the FTD and HD-MA groups, respectively (*P* = 0.509), with a median OS of 48.8 months (95% CI 24.2–73.4) versus 44.9 months (95% CI 18.7–71.2), respectively (Fig. 2b). No significant differences were recorded for either PFS or OS in patients from the two treatment groups.

**Table 3:** Response of the FTD and HD-MA regimens

Responses status	Number of patients (%)		*P* value
	FTD (n = 24)	HD-MA (n = 25)	
CR	8 (33)	10 (40)	0.628
PR	13 (54)	11 (44)	0.571
ORR	21 (88)	21 (84)	0.726
SD	0 (0)	2 (8)	NA
PD	3 (12)	2 (8)	0.603

*CR* complete response, *PR* partial response, *SD* stable disease, *PD* progressive disease, *ORR* overall response rate, *NA* not applicable

*P*: Chi square test

### Toxicity

Adverse reactions were assessed in all eligible patients, including hematological and non-hematological toxicities (Table 4). The most common adverse reactions to both regimens were hematological toxicities signifying myelosuppression, including leucopenia, thrombocytopenia and anemia. Most non-hematological toxicities were mild and transient, including digestive tract toxicity and electrolyte imbalance. The HD-MA group showed more serious neutropenia (*P* = 0.009) and hepatic dysfunction (*P* = 0.010) than the FTD group. Grade 3–4 hematological toxic effects were increased in the HD-MA group compared with those in the FTD group. Methotrexate dose reduction due to toxicity was reported in 5 (20%) patients in the HD-MA group and 1 (4%) patients in the FTD group. In addition, one case underwent grade 4 infection, which led to patient death in the HD-MA arm.

**Table 4:** Main adverse effects between FTD and HD-MA group

Toxicity	Grade of adverse reaction							*P* value
	FTD (n = 24)				HD-MA (n = 25)			
Grade	0	1–2		3–4	0	1–2	3–4	
Hematologic								
Leukopenia	11	6		7	3	7	15	0.009
Neutropenia	13	5		6	4	8	13	0.009
Anemia	8	14		2	5	12	8	0.064
Thrombocytopenia	12	6		6	7	9	9	0.161
Non-hematologic								
Infection	17		7	0	15	7	3	0.303
Digestive tract toxicity	17		6	1	15	7	3	0.365
Mucositis	23		1	0	20	4	1	0.091
Electrolyte imbalance	17		6	1	17	6	2	0.776
Hepatic dysfunction	16		7	1	7	16	2	0.010
Renal dysfunction	24		0	0	22	3	0	0.083
Cardiac toxicity	23		1	0	21	1	3	0.159

*P*: Mann–Whitney test

Of these 49 patients, 7 had achieved CR on first-line chemotherapy without WBRT (older than 60 years); 42 with first-line chemotherapy followed by WBRT, of whom 7 patients received rescue WBRT for SD or PD during chemotherapy (3 in the FTD group, and 4 in the HD-MA group). Delayed neurotoxicity was assessed in 23 patients after median follow-up of 31 months (range 11.2–69.0 months) and was recorded in 3 (23%) of 13 patients receiving FTD followed by WBRT and in 6 (60%) of 10 of those receiving HD-MA followed by WBRT.

### Molecular prognostic variables

Diagnostic specimens were requested from all participating patients, and sufficient biopsy material was available for immunohistochemical staining from the patients. The frequencies of expression for Bcl-2, Bcl-6, c-Myc, MUM-1, CD5, CD10, PTEN and p53 were 61%, 65%, 57%, 73%, 59%, 33%, 53% and 55%, respectively. 17 of the 49 patients (35%) showed high tumor cell proliferation (≥ 90%). The univariate analysis suggested that the expression of Bcl-6 (Fig. 2c, *P* = 0.038) was related to longer OS. Median OS was significantly longer in patients with Bcl-6 expression positive (58.4 months, 95% CI 45.4–73.1) than those Bcl-6 expression negative (23.9 months, 95% CI 3.2–44.7). In multivariate regression analyses that included Bcl-2, Bcl-6, c-Myc and CD10, with OS as the dependent variables, only Bcl-6 expression reached statistical significance (RR = 2.539; *P* = 0.039) as an independent predictor of OS in PCNSL.

## Discussion

PCNSL has posed a major challenge to physicians for decades. To date, this is the first prospective randomized clinical trial comparing the efficacy, toxicity and survival analysis of FTD versus HD-MA regimens followed by WBRT in newly diagnosed PCNSL. The results of this pilot study demonstrate that it could be a valid alternative to build-up gradually an active chemotherapy combination for this lymphoma.

In this study, we have selected a combination of chemotherapeutic drugs based on their pharmacokinetic properties and dose levels aimed at delivering effective chemotherapy to the CNS. Fotemustine (Muphoran) is an anti-cancer drug from the family of nitrosourea, which are known to penetrate into the CNS, and experimentally it has a large anti-tumour activity. The drug has been approved in the treatment of malignant metastatic melanoma and primary brain tumors [18]. Teniposide is a potent inhibitor of topoisomerase II. The EORTC (European Organization for Research and Treatment of Cancer Lymphoma Group) Phase II trial 20962 indicated that the application of methotrexate, teniposide, carmostine and methylprednisolone resulted in improved outcomes with a 3-year OS rate of 58% [9]. Although this study used multiple drugs, the results may indicate that teniposide is an effective drug for lymphoma. Although no significant differences were recorded for either ORR or outcomes in patients from both treatment groups, the 88% ORR in the FTD group demonstrate the efficacy of FTD for newly diagnosed PCNSL patients. And, we recommend FTD as an alternative to HD-MA in patients who cannot take risk of side effects. It is possible that with larger sample size and longer follow-up differences in outcome may emerge.

HD-MTX-based polychemotherapy followed by WBRT plays a central role in the management of PCNSL [19]. A phase 3 randomized non-inferiority trial (G-PCNSL-SG-1) concluded that WBRT can be omitted from the first-line treatment of PCNSL without compromising OS, however, the data demonstrated that patients who received WBRT had a significantly longer PFS of 18 months compared with those who did not receive WBRT (12 months) [16]. Delayed neurotoxicity is a major concern with combination chemotherapy and radiotherapy, particularly in patients over 60 years of age [20, 21]. For patients achieving CR with chemotherapy, reduction of the dose of WBRT was reported to compromise survival and minimal neurotoxicity in a phase 2 study [22]. In our study, as defined in the study protocol, patients aged 60 years or younger in CR were treated with 30 Gy consolidating WBRT; those older than 60 years in CR were treated with watchful waiting. This could be clinically relevant in lower incidence of neurotoxicity in our study.

The particular molecular prognostic biomarkers have not been clearly identified for PCNSL. In our study, Bcl-6 expression was associated with a statistically significant survival advantage compared with tumors that did not express the protein, consistent with previous studies showing significantly better survival associated with expression of the Bcl-6 oncoprotein [23–25]. However, the available data are conflicting. Two other studies have found that the expression of Bcl-6 was correlated with adverse outcomes in PCNSL [26, 27]. Table 5 list details of baseline evaluation, sample size, cut-off level, and *P*-value reported in these articles. Possible explanations for these disparate findings are the small number of tumours analyzed in the majority of these studies. Although our study has evaluated the significance of Bcl-6 expression in PCNSL, more prospective controlled trials must be conducted given the small size and number of biopsy specimens available, with consequent limited power.

**Table 5:** Baseline evaluation in published reports

References	Sample size	Cut-off level (%)	No. of patients (%)	*P* value
Levy et al. [23]	57	50	26 (46)	0.18
Lossos et al. [24]	69	30	39 (56)	0.055
Lin et al. [25]	29	20	19 (66)	0.073
Rubenstein et al. [26]	26	30	19 (73)	0.045
Momota et al. [27]	27	30	13 (48)	0.038

Several limitations of the current trial must be considered. First, limitations include the limited number of cases and the censored data in the subgroups for detailed analysis due to the lower morbidity and higher mortality of PCNSL. However, We choosed HD-MA regimen as the control group whose efficacy has been repeatedly confirmed by prospective trials [5]. The median OS was 44.9 months in our HD-MA group, consistent with previous researches [2–4], demonstrating that our control group was reliable. Second, the effect of treatment on neurocognitive functions was assessed by Mini-Mental State Examination (MMSE) and a panel of neuro psychological tests presently used by the International PCNSL Collaborative Group (IPCG) [28]. However, the neurotoxicity analysis maybe weakened in our study by the small sample size. Specifically, rituximab, a CD20-targeted monoclonal antibody, has demonstrated promising effects in PCNSL [29, 30]. Despite these limitations, our data indicate that the FTD regimen appears to be beneficial to patients.

In conclusion, this study has identified, for the first time, an FTD regimen that is an effective and safe protocol for treating patients with newly diagnosed PCNSL. However, further studies are required to confirm our observations.
